# Improvement of diabetic wound healing by topical application of Vicenin-2 hydrocolloid film on Sprague Dawley rats

**DOI:** 10.1186/s12906-018-2427-y

**Published:** 2019-01-17

**Authors:** Woan Sean Tan, Palanisamy Arulselvan, Shiow-Fern Ng, Che Norma Mat Taib, Murni Nazira Sarian, Sharida Fakurazi

**Affiliations:** 1 Laboratory of Vaccines and Immunotherapeutics, Institute of Bioscience, Universiti Putra Malaysia, 43400 Serdang, Selangor Malaysia; 2 Centre of Drug Delivery Research, Faculty of Pharmacy, Universiti Kebangsaan Malaysia, Jalan Raja Muda Abdul Aziz, 50300 Kuala Lumpur, Malaysia; 3 Department of Human Anatomy, Faculty of Medicine and Health Sciences, Universiti Putra Malaysia, 43400 Serdang, Selangor Malaysia

**Keywords:** Vicenin-2, Sodium alginate, Hydrocolloid film, Diabetic wound

## Abstract

**Background:**

Impaired wound healing is a debilitating complication of diabetes that leads to significant morbidity, particularly foot ulcers. The risk of developing diabetic foot ulcers for diabetic patients is 15% over their lifetime and approximately 85% of limb amputations is caused by non–healing ulcers. Unhealed, gangrenous wounds destroy the structural integrity of the skin, which acts as a protective barrier that prevents the invasion of external noxious agents into the body. Vicenin-2 (VCN-2) has been reported to contain prospective anti-oxidant and anti-inflammatory properties that enhance cell proliferation and migration. Sodium Alginate (SA) is a natural polysaccharide that possesses gel forming properties and has biodegradable and biocompatible characteristics. Therefore, the objective of this study is to evaluate the effect of SA wound dressings containing VCN-2 on diabetic wounds.

**Methods:**

Wounds were inflicted in type-1 diabetic-streptozotocin (STZ) induced male Sprague Dawley rats. Subsequently, relevant groups were topically treated with the indicated concentrations (12.5, 25 and 50 μM) of VCN-2 hydrocolloid film over the study duration (14 days). The control group was treated with vehicle dressing (blank or allantoin). Wounded tissues and blood serum were collected on 0, 7 and 14 days prior to sacrifice. Appropriate wound assessments such as histological tests, nitric oxide assays, enzyme-linked immunosorbent assays (ELISA) and immunoblotting assays were conducted to confirm wound healing efficacy in the in vivo model. One-way Analysis of Variance (ANOVA) was used for statistical analysis.

**Results:**

Results showed that hydrocolloid film was recapitulated with VCN-2 enhanced diabetic wound healing in a dose-dependent manner. VCN-2 reduced pro-inflammatory cytokines (IL-1β, IL-6 and TNF-α), mediators (iNOS and COX-2), and nitric oxide (NO) via the NF-κB pathway. Data suggests that the VCN-2 film facilitated healing in hyperglycemic conditions by releasing growth factors such as (VEGF and TGF-β) to enhance cell proliferation, migration, and wound contraction via the VEGF and TGF-β mechanism pathways.

**Conclusions:**

This study’s findings suggest that VCN-2 may possess wound healing potential since topical treatment with VCN-2 hydrocolloid films effectively enhanced wound healing in hyperglycemic conditions.

## Background

Wound healing is a natural process that involves a cascade of complicated cellular and biomolecular processes that restore damaged wound tissue into its original state when injury occurred. The fundamental biological wound repair process is comprised of inflammation, cell migration/cell proliferation, and remodelling. The orderly progression of healing events leads to rapid wound closure, and for acute wound healing a minimal or aesthetically acceptable scar with no regeneration [1]. This process takes place at an optimal rate in healthy individuals, but it is usually delayed or impaired for patients in diabetic conditions.

Altered wound healing is one of the most common complications in Diabetes Mellitus (DM). The wound healing process in patients with DM is deteriorated due to hyperglycaemic conditions that lead to major chronic complications, such as Diabetic Foot Ulcers (DFUs) [2]. Abnormal wound healing often leads to chronic ulcer formation, which has caused major worldwide morbidity due to various clinical and socioeconomic issues. According to Brem and Canic [3], this is a 15% risk of getting foot ulcers for people with diabetes, of which 85% will have to undergo lower extremity amputations.

The mechanism of delayed wound healing is multifactorial, including a prolonged inflammatory stage and postponed proliferation and remodelling stages [4]. DeClue and Shornick [5] reported that diabetic wound healing was associated with the over release of proinflammatory cytokines such as IL-1β, IL-6, and TNF-α. Qiu et al. [6] revealed that diabetic patients with high blood glucose showed hindered cell proliferation and declined production of collagen and growth factors during wound healing. Reduced angiogenesis is also an important cause of impaired wound healing in diabetics with low levels of the VEGF and TGF-1β growth factors [7]. In this study, these biomarkers were selected to measure drug mechanisms during wound healing.

There have been various attempts to accelerate wound healing in diabetics, but so far only a few effective therapeutic remedies are available. Alternative therapeutic treatments using natural products are highly demanded. The Vicenin-2 (VCN-2) active compound in plants was targeted for diabetic wound healing efficacy due to its antidiabetic [8], anti-hyperglycemic [9, 10], anti-oxidant [11], anti-inflammatory [12, 13], cell proliferation, and cell migration activities [14].

Dressings cast from hydrocolloid films are a popular wound dressing owing to their high absorption ability via strong hydrophilic gel formation [15]. Furthermore, alginate is a natural polysaccharide found in the cell walls of brown algae that possesses high gel forming properties that are biodegradable and biocompatible in nature [16]. Sodium alginate (SA) is usually exploited as a drug-controlled release vehicle in drug delivery systems due to the various cross-linking polymers present in it. Sodium Alginate (SA), which provide reduce hydrocolloid film swelling from water exposure (wound exudate). This is important in controlling the slow release properties of bioactive materials (i.e. drugs) from the gel and ensuring high formulation efficacy [17].

This goal of this study was to investigate the efficacy of a new formulation of hydrocolloid dressing, a Sodium Alginate (SA) biomaterial scaffold with VCN-2, on the healing of excision wounds in a type-1 diabetic Sprague Dawley rat. This study suggests that VCN-2 hydrocolloid film hold considerable promise in diabetic wound healing, and is a potential therapeutic dressing for enhanced diabetic skin wound healing in the future.

## Methodology

### Chemical and reagents

Commercial standard Vicenin-2 (VCN-2) was purchased from Haihang Industrial Company (Beijing, China). Allantoin brand Fluka Analytical and Streptozotocin (STZ) were obtained from Sigma Aldrich Chemicals (St. Louis, MO, USA). Diagnostic kits for VEGF and TGF-β were bought from Bender MedSystems (Vienna, Austria) while insulin diagnostic kits were acquired from Mercodia Corporation (Uppsala, Sweden). RIPA buffer and sulphanilamide were received from Nacalai (Kyoto, Japan) and Friedman Schmidt (CT Parkwood, WA, Australia), respectively. The standard protein assay kit (Pierce™ 660 nm Protein Assay) and bicinchoninic acid (BCA) assay were bought from Thermo Fisher Scientific (Waltham, MA, USA). Primary antibodies specific to iNOS, COX-2, NF-휅B, MMP-9, VEGF, TGF-*β*, *β*-actin, and anti-rabbit and/or anti-mouse secondary antibodies conjugated into horseradish peroxidase were attained from Santa Cruz Biotechnology (Santa Cruz, CA). All the chemicals used in the experiments were of an analytical grade.

### Animal models

In this study, 84 healthy male adult Sprague Dawley rats (7–8 weeks old), weighing between 150 and 250 g, were kept in polypropylene cages at the animal house of the Faculty of Medicine and Health Sciences, Universiti Putra Malaysia. The animals were maintained under standard conditions: temperature (25 ± 2 °C), relative room humidity (55%) and alternating light-dark cycle (12 h/12 h), with access to standard pellets and water ad libitum for the entire experimental period. The animal experiments were approved by the Institutional Animal Care and Use Committee (IACUC) of Universiti Putra Malaysia (authorization number UPM/IACUC/AUP-R079/2016) in accordance with the recommendations for handling animals for research. In this experiment, rats were divided into six groups on day 7 and day 14, with each group having six animals. On day 0, rats were only divided into normal and diabetes groups (*n* = 6).

### Induction of diabetes

To induce the diabetes mellitus experimental model, 66 animals were injected via single intraperitoneal injection with STZ (50 mg/kg) freshly prepared in a cold 0.1 M sodium citrate buffer (pH 4.5) prior to fasting for approximately 16 h, with only water provided ad libitum. The rats in the normal group were injected intraperitoneally with sodium citrate buffer (pH 4.5) [18, 19]. Three days after induction, a drop of blood (~ 1.5 μl) from the tail vein of each rat was obtained using a lancet. The blood was tested using a commercial glucometer (ACCU-CHECK Active Glucose Monitor, Roche, Germany) to determine hyperglycemia development. The fasting rats with a blood glucose level > 11 mmol/L on day 7 after injection were considered diabetic and were used in further experiments in this study [20].

### Induction of wound

All animals were anaesthetised with 90 mg/kg ketamine and 10 mg/kg xylazine intraperitoneally following confirmation of diabetes. Prior to wound infliction, the dorsal fur of the animals was shaved with an electric clipper and disinfected with 70% ethanol. A standardized full thickness open excision wound was created using a biopsy punch (Cat no.12–460-412, Thermo Fischer Scientific, USA) that was 6 mm in diameter and 2 mm in depth [21]. The wound was left uncovered for 24 h. Rats with an open wound were kept in a polypropylene cage.

### Treatment of wound

Various concentrations of formulated VCN-2 hydrocolloid film was applied topically to the rats every day for 14 days, beginning 24 h after the wound was inflicted. Based on Table 1, there were 6 animal treatment groups: normal rats treated with blank film (Group I), diabetic rats treated with blank film (Group II), diabetic rats treated with 12.5, 25, and 50 μM VCN-2 film (Group III-V); and diabetic rats treated with 316 μM allantoin film (Group VI). Wounds were treated by applying a 0.8 cm^2^ film dressing and adhesive-permeable bandage wrapping. Allantoin film was used as the positive drug control for this experiment.

**Table 1:** Division of animal according to groups

No	Group	Quantity of rat	Description Treatment
I	Normal	18	Normal rat (N) + Wound (W) + Blank film
II	Diabetic	18	Diabetic rat (D) + W + Blank film
III	12.5 μM VCN-2	12	D + W + 12.5 μM VCN-2 film
IV	25 μM VCN-2	12	D + W + 25 μM VCN-2 film
V	50 μM VCN-2	12	D + W + 50 μM VCN-2 film
VI	316 μM ALN	12	D + W + 316 μM ALC film

Abbreviations: *VCN-2* Vicenin-2, *ALN* allantoin, *N* normal rat, *D* diabetic rat and *W* wound

### Determination of blood glucose, insulin level and measurement of body weight, pellet and fluid intake

Biochemical determinations (blood glucose and insulin level) and physiological measurements (body weight, pellet and fluid intake) were measured on day 7 and day 14 for all groups. Blood glucose readings for each rat was obtained using a glucometer. The harvested animal group blood samples were collected via cardiac puncture and clotted at room temperature for 15 to 30 min, before being centrifuged at 2200 to 2500 rpm for 15 min to create a blood serum. A quantitative determination of serum insulin for all the rats was done using a rat insulin ELISA kit in accordance with manufacturer instructions. Body weight as well as pellet and fluid intake for all animal groups was monitored on a daily basis for 2 weeks using a balance and measuring cylinder. Fixed amounts of standard pellets and fluids were given to the rats and both were replenished on the next day.

### Gross evaluation of wound contraction

Reductions in wound area over time has been reported to predict wound healing in diabetic foot ulcers. Wound areas on day 7 and 14 are regarded as an important indicator of wound healing [22]. Animal wounds were evaluated macroscopically on day 0, 7, and 14 to assess the healing process and to measure the wound area. Wound closure rates were expressed as a percentage of original wound area. The wound was photographed with a digital camera to judge wound closure progress. Due to the irregular shapes of the wounds (circular), outer wound margins in this study were traced on a transparent sheet using a permanent marker. The planimetrical surface area was measured on graph paper according to the method assessed by Ponrasu and Suguna [23]. According to Lin et al. [24], the percentage (%) of the wound contraction was then determined using the formula below:

$$ \boldsymbol{Wound}\kern0.17em \boldsymbol{contraction}\kern0.17em \boldsymbol{rate}\;\left(\%\right)=\frac{\boldsymbol{Initial}\kern0.17em \boldsymbol{wound}\kern0.17em \boldsymbol{size}-\boldsymbol{wound}\kern0.17em \boldsymbol{size}\;\boldsymbol{on}\;\boldsymbol{specific}\;\boldsymbol{day}\;\left(\boldsymbol{mm}\mathbf{2}\right)}{\boldsymbol{Initial}\kern0.17em \boldsymbol{wound}\kern0.17em \boldsymbol{size}\;\left(\boldsymbol{mm}\mathbf{2}\right)}\boldsymbol{x}\mathbf{100}\% $$

### Histological study of wound

Prior to animal sacrificed and wound tissue harvested, rats were euthanized by cervical dislocation under anesthesia using diethyl ether. Histological examinations were carried out to assess cellular responses and vascularisation of wound tissue for further wound healing evaluation. Biopsies of wounded areas were performed using histological evaluations 7 and 14 days post-wounding. The wound samples were fixed with a 10% formaldehyde buffer solution for 72 h. Samples then underwent a standard dehydration process in a series of increasing ethanol concentrations for 24 h by a processing machine. Then, the tissue was degreased with xylene, embedded in paraffin, and sectioned using a histological microtome. Sections of 5 μm thick tissue were mounted on a glass slide and stained using haematoxylin and eosin (H&E). This was followed by the visualisation of samples under a light microscope at 100 x and 400 x magnification.

A semi-quantitative method was used to examine the following histological structures and processes: epidermal regeneration (100 x), granulation tissue thickness (100 x), fibroblast proliferation (400 x), angiogenesis (400 x) and the presence of inflammatory cells (400 x). Three stained sections from 10 random fields for each group were evaluated and scored using a scale of 1–3 for epidermal regeneration and granulation tissue thickness; and a scale of 0–4 for fibroblast proliferation, angiogenesis, and the presence of inflammatory cells as summarized in Table 2. All stained images were taken by using an Olympus microscope (BX-51; Olympus, Tokyo, Japan).

**Table 2:** Histological features of wound healing

Histological score	0	1	2	3	4
Epidermal Regeneration	None	Mild	Moderate	Complete	–
Granulation tissue thickness	None	Mild	Moderate	Complete	–
Fibroblast proliferation	Absence	Occasional presence	Light scattering	Abundance	Confluence
Presence of neovascularization (angiogenesis)	Absence	Occasional presence	Light scattering	Abundance	Confluence
Presence of inflammatory cells	Absence	Occasional presence	Light scattering	Abundance	Confluence

(A modification of Altavilla et al. [25], Lee et al. [26] and Gopalakrishnan et al. [27])

### Examination of NO production of wound through NO screening assay

One gram of wound tissue from each group was cut into pieces using a scalpel/surgical blade, and then pulverized with a mortar/pestle following tissue sample collection as described in Table 1. Under ice conditions, 3 mL RIPA buffer was added into a 10 mL centrifuge tube contained skin tissue for homogenization. Skin tissue samples were incubated on ice for 2 h, after which the homogenates were centrifuged at 10000 X g for 10 min at 4 °C. Wound lysates (supernatants) containing whole proteins was transferred into a new microtube and stored at − 20 °C for experimental analysis. To measure nitrite levels, the supernatants of wound lysates with 100 μL were mixed with 100 μL freshly prepared Griess reagent (0.1% N-(1-naphthyl) ethylenediamine dihydrochloride, 1% sulphanilamide, and 2.5% phosphoric acid). The mixture solution was incubated for 10 min and absorbance was measured at 540 nm using a microplate reader. Sodium nitrate (NaNO_2_) was compared with the standard curve.

### Measurement of cytokines (IL-1β, IL-6 and TNF-α) and growth factors (VEGF and TGF-β) productions of wound through ELISA

The concentration of proinflammatory cytokines, including IL-6, IL-1 β, and TNF-α as well as healing growth factors such as VEGF and TGF-β, were detected according to manufacturer instruction using DuoSet ELISA Development (R&D Systems, MN, USA). Basically, selected standard and wound lysate samples were pipetted into antibody coated wells and incubated with assay diluents for 2 h at room temperature. The wells were then washed 5 times with a wash buffer, before being incubated with a conjugate solution for 2 h. This step was repeated for incubation with streptavidin-HRP for 1 h. TMB substrate solution with 100 μL was added into each well and allowed to stand for 30 min following the five-time washing process. Finally, 50 μL stop solution was added to stop enzyme reactions. Optical density was read using a microplate reader (BioTek Instruments Inc., VT, USA) at a 450 nm wavelength with 570 nm cytokine and 620 nm growth factor references. Protein concentrations were calculated from the standard curve.

### Detection of mediators (iNOS, COX-2, NF-κB, MMP-9, anti-HIF, VEGF and TGF-β) expression of wound through Western blot

The total protein of wound lysates was determined using a Bradford reagent assay, with bovine serum albumin reagents acting as a standard. The 10 μL of each sample and standard was added to 96 well plates and mixed with 150 μL of protein assay solution, followed by 10 min incubation. The absorbance of reaction products was measured at a 600 nm wavelength using a microplate reader. An equal amount of protein (20 μg/mL) was resolved on Sodium Dodecyl Sulfate (SDS) gels using SDS-polyacrylamide gel electrophoresis (PAGE) before being transferred to PDVF membranes. Nonspecific membrane sites were then blocked with 5% BSA, followed by primary antibody incubation (1:1000 dilution) of iNOS, COX-2, NF-κB, MMP-9, anti-HIF, VEGF, TGF-fβ, and β-actin at 4 °C overnight. Corresponding anti-rabbit, anti-goat, or anti-mouse secondary antibodies (1:5000 dilution) were conjugated with horseradish peroxidase for 1 h. Membranes were detected using a Super Signal West Femto Chemiluminescent Substrate (Thermo Scientific) and analysed using Image J software.

### Statistically analysis

Experiment data is presented as mean ± Standard Error Mean (SEM) in this study. The differences between various means were calculated using IBM with SPSS 20.0 software (SPSS Inc., Chicago, USA). One-Way Analysis of Variance (ANOVA) was used to compare groups, and Tukey post-hoc tests were used for pairwise comparisons following normality and homogeneity tests. A *p-*value of 0.05 or less was considered to be statistically significant.

## Result

### Biochemical determination of blood glucose level in rats

Blood glucose levels were elevated in rats administrated with STZ. Blood glucose levels for the normal rat control group were 5 mmol/L, and > 20 mmol/L for the diabetic rats. In this study, the blood glucose levels of diabetic rat were more than 11 mmol/L throughout the duration of the experiment. From day 7 to day 14, blood glucose decreased following topical application of a blank film or VCN-2 containing film for the diabetic group. However, the blood glucose in diabetic group was found higher than the normal group after they were treated with a blank film (Table 3).

**Table 3:** Total blood glucose, insulin level, body weight, food intake and water consumption of treatment groups of rats on day 7 and 14

Group Treatment	Blood glucose (mmol/L)		Insulin concentration (pg/mL)		Body weight (g)		Food intake (g)		Water consumption (mL)	
	Day 7	Day 14	Day 7	Day 14	Day 7	Day 14	Day 7	Day 14	Day 7	Day 14
Normal	5.2 ± 0.7	4.9 ± 0.1	40.5 ± 4.4	46.7 ± 7.1	280.3 ± 18.8	312.7 ± 17	130.5 ± 14.4	193.9 ± 4.0	246.6 ± 5.8	393.0 ± 3.7
Diabetic	22.2 ± 1.2	20.7 ± 2.4	5.1 ± 0.1	7.1 ± 0.8	235.0 ± 22.5	208.7 ± 3.3	152.6 ± 2.3	260.6 ± 10.0	617.9 ± 14.4	967.9 ± 9.5
VCN-2 film										
12.5 μM	17.1 ± 0.2	14.4 ± 2.7	12.8 ± 3.1	27.4 ± 0.6	253.4 ± 9.7	268.7 ± 3.4	144.8 ± 11.5	247.0 ± 9.6	598.6 ± 17.3	927.2 ± 17.3
25 μM	16.0 ± 0.3	14.4 ± 1.0	21.5 ± 0.3	27.7 ± 0.9	251.0 ± 25.5	277.7 ± 2.6	140.0 ± 5.8	243.6 ± 11.5	589.3 ± 13.2	867.9 ± 13.2
50 μM	21.1 ± 1.7	15.3 ± 3.3	23.2 ± 3.0	31.3 ± 4.3	260.2 ± 31.5	280.2 ± 3.5	133.3 ± 12.6	235.4 ± 14.5	574.3 ± 14.4	845.7 ± 28.8
ALN film										
316 μM	20.7 ± 1.6	14.0 ± 1.3	29.2 ± 3.8	30.7 ± 4.0	236.0 ± 30.7	248.8 ± 5.8	139.4 ± 8.7	245.1 ± 14.4	584.3 ± 28.8	862.8 ± 25.1

Abbreviations: *VCN-2* Vicenin-2, *ALN* allantoin

### Biochemical analysis of insulin level in rats

Table 3 shows that blood insulin levels from the normal group were 40.5 ± 4.4 pg/mL on day 7 and 46.7 ± 7.1 pg/mL on day 14. In diabetic animals topically treated with blank film and VCN-2, insulin levels were lower on day 7 than on day 14. Even though the concentration of insulin was observed to increase in diabetic animals treated with VCN-2 film, it was significantly lower than the normal control group treated with blank film.

### Physiological measurement of body weight in rats

As shown in Table 3, there was a reduction in body weight for rats in the diabetic group (208.7 ± 3.3 g) as compared to the normal control group (312.7 ± 17.0 g) by day 14. In contrast to STZ induced diabetic rats, the normal control rats had a persistently elevated body weight during the duration of this study. In diabetic rats treated with VCN-2, body weight was increased in a dose dependent manner. On day 14, diabetic rat body weights were 268.7 ± 3.4 g, 277.7 ± 2.6 g and 280.2 ± 3.5 g for those treated with 12.5, 25 and 50 μM of VCN-2 hydrocolloid films, respectively. Nonetheless, the body weight of the diabetic rats was lower than the normal group.

### Quantification of food and fluid intake of rats

Table 3 indicates the total food and water intake of the rats during the experiment. Diabetic rats clearly consumed more food than the normal control group. Diabetic rats treated with VCN-2 and allantoin hydrocolloid films consumed a slightly higher quantity of food. There was a rise in water intake for the rats in diabetic conditions over the rats in normal conditions. Table 3 elucidates total rat water consumption data 967.9 ± 9.5 mL (diabetic control) and 393.0 ± 3.7 mL (normal control) on day 14.

### Effect of VCN-2 hydrocolloid film on wound contraction

**Fig. 1:**
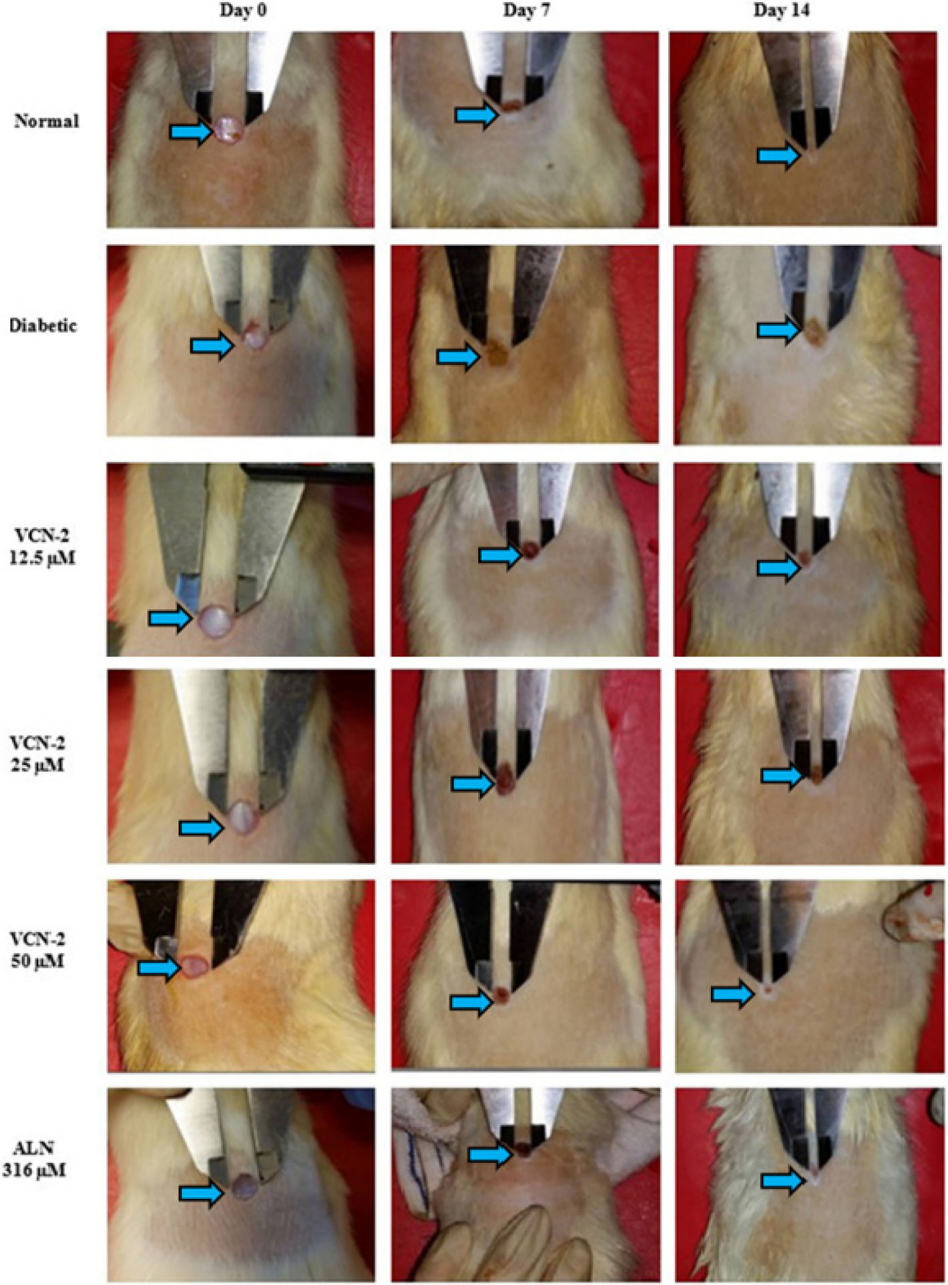
Representative image of wounds of the normal control, diabetic control, 12.5, 25, 50 μM VCN-2 and 316 μM allantoin treated groups on day 0, 7 and 14. Result shows treatment with VCN-2 enhanced wound healing in diabetic rats as compared to diabetic rats treated with blank film (*n* = 6)

Figure 1 displays the effect of VCN-2 hydrocolloid films on excised wound contractions in diabetic rats. Results showed that rats treated with VCN-2 film presented faster and higher re-epithelialization in the in vivo full thickness excisional wounds created on the rats dorsal backs. This result was compared with normal control rats treated with a blank dressing. Based on Table 4, the percentage of wound contraction in diabetic rats was significantly lower than the normal control (*p* < 0.001). On day 7, the wound percentage for the normal control was 30.02 ± 1.74% and the diabetic control was 14.94 ± 2.51%. On day 14, the wound percentage for the normal and diabetic control was 62.20 ± 1.31% and 27.27 ± 2.27%, respectively. Diabetic rat groups treated with VCN-2 films showed noticeable wound contraction in a concentration dependent manner. Treatments with 50 μM VCN-2 film showed the highest wound contraction percentage (39.91 ± 3.17% on day 7 and 63.06 ± 1.94% on day 14). The positive control rats treated with allantoin film expressed good recovery results with a wound contraction percentage of (59.35 ± 2.03%) throughout 14 days of treatment.

**Table 4:** Percentage of the wound contraction in rats with post infliction of excised wound after treatment for 14 day

No	Group Treatment	Day 7 (%)	Day 14 (%)
I	Normal	30.02 ± 1.74	62.20 ± 1.31
II	Diabetes	14.95 ± 2.51^###^	27.27 ± 2.27^##^
III	12.5 μM VCN-2 film	23.58 ± 0.80**	43.36 ± 3.39**
IV	25 μM VCN-2 film	28.47 ± 6.84**	56.94 ± 3.67***
V	50 μM VCN-2 film	39.91 ± 3.17***	63.06 ± 1.94***
VI	316 μM ALN film	31.02 ± 4.56***	59.35 ± 2.03***

The duration of treatments was 14 days. Values (*n* = 6) represent a mean ± SEM. ^###^*p* < 0.001 and ^##^*p* < 0.01, diabetic control group vs normal control group; ***p < 0.001 and **p < 0.01 treatments groups vs diabetic control group

### Histological evaluation of wound tissue for epithelialization and granulation formation

**Fig. 2:**
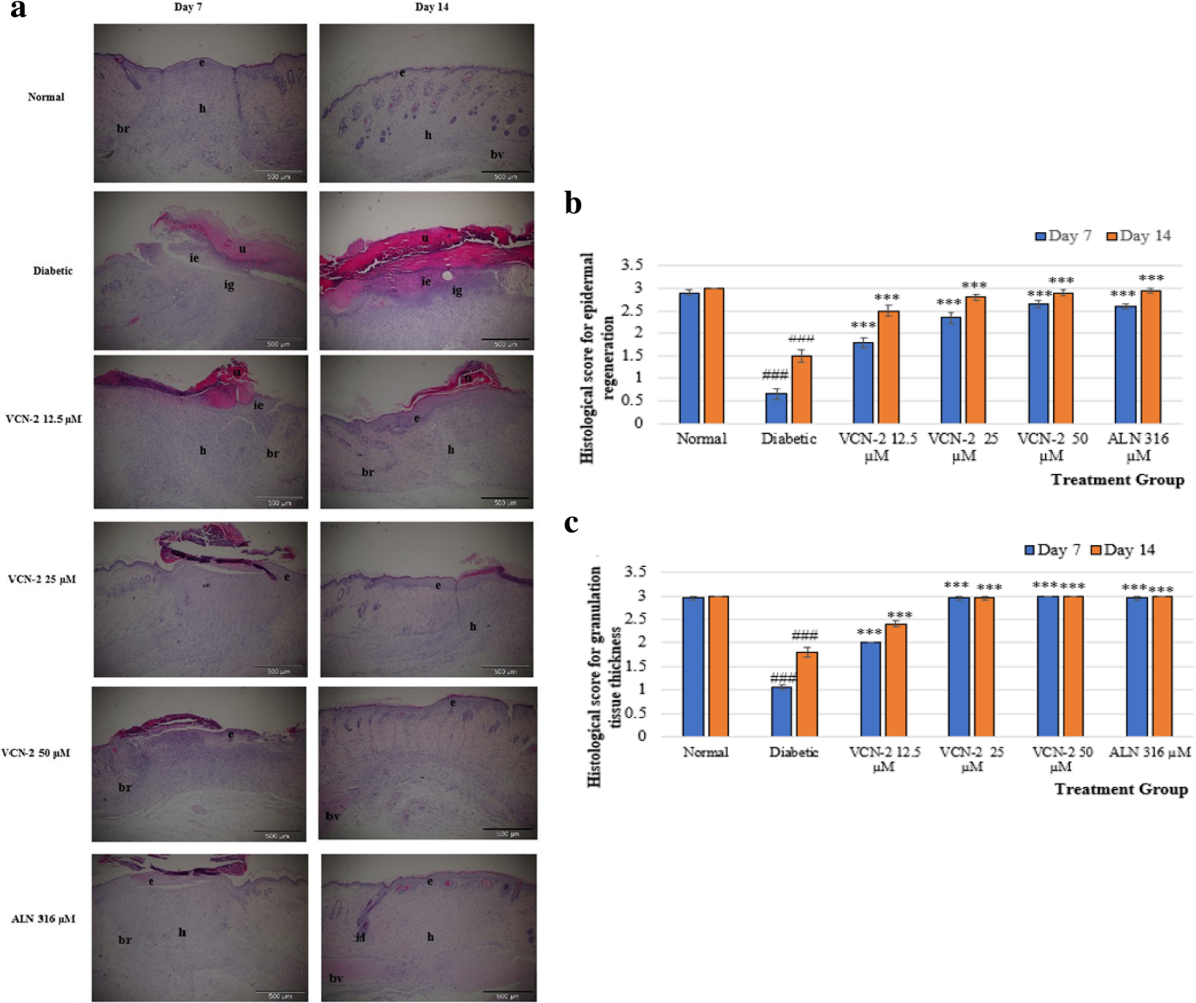
**a** Images of healed wound site from a normal rat and diabetic rat with blank, VCN-2 and allantoin dressing treatment on day 7 and 14. Normal rat demonstrating complete re-epithelialization and well-form granulation tissue. Diabetic rat treated with VCN-2 12.5, 25 and 50 μM film consequence re-epithelialization and well-formed granulation tissues on day 7 and 14, (100x with H&E stain). Abbreviation (br): boundary between unhealed and healed tissue; (bv): blood vessels; (h): healed area with multiple layers of fibrous connective tissue; (e): epithelium; ie): immature epidermis; (ig): immature granulation tissue and (u): ulcer. **b** Histologic scoring for epidermal regeneration. **c** Histologic scoring for granulation tissues thickness of the rats on day 7 and 14 from 10 randomly chosen fields. Data expressed as means ± SEM (*n* = 6). ### *p* < 0.001 is diabetic group compared with normal group; *** *p* < 0.001 is treated group compared with diabetic group on the same day

In this study, histological evaluations were conducted to study the response of cellular wound repair mechanisms from the use of VCN-2 hydrocolloid dressings on diabetic Sprague Dawley rats. Figure 2(a) shows histological observations for skin epidermal regeneration and granulation tissue thickness on day 7 and 14. Results in Fig. 2 (a) suggest that blank film treated diabetic rats showed incomplete reepithelialisation and poorly formed granulation tissue, but the VCN-2 film treated diabetic group and blank film treated normal group show moderate reepithelialisation with well-formed granulation tissue after 14 days of treatment. Topical application of VCN-2 film steadily enhanced epidermal regeneration (2(b)) and granulation tissue thickness (2(c)) in a dose dependent manner.

### Histological analysis of wound tissues for fibroblast proliferation, angiogenesis and presence of inflammatory cells

**Fig. 3:**
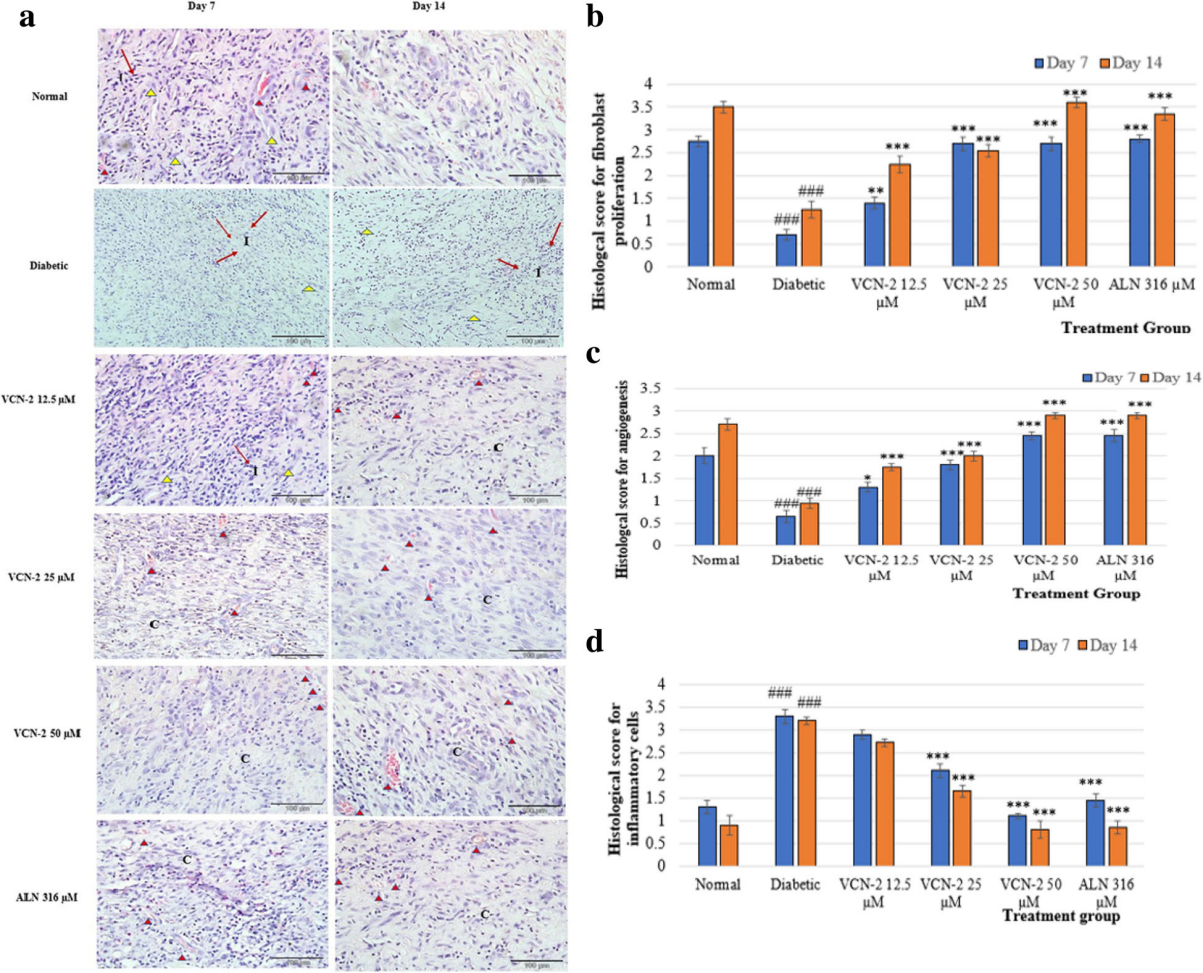
**a** Normal rat and diabetic rat treated with blank, VCN-2 and allantoin dressing on day 7 and 14. Diabetic rats treated with VCN-2 showed more neovascularization on day 14 compared to day 7. Wound of rat on day 14 contained more blood vessels and showed well connective tissue deposition, *n* = 6, 400x with H&E staining. Abbreviations: (I): Inflammatory cell and (C): Connective tissue. Representation: angiogenesis and : fibroblast cell. **b** Histological finding score of fibroblast proliferation; (**c**) angiogenesis and (**d**) inflammatory cells infiltrate (score from 0 to 4) from 10 randomly chosen high-power fields (× 400) from three sections in wound treated with difference films on day 7 and 14. Data are means ± SEM histological score (*n* = 6). ###*p* < 0.001, diabetes versus normal; *p < 0.05, **p < 0.01 and ***p < 0.001, treatment groups versus diabetic

The rate of proliferation for the fibroblast and angiogenesis as well as the population of inflammatory cells is depicted in Fig. 3(a). Results suggest that animals treated with 50 μM VCN-2 and 316 μM allantoin hydrocolloid films showed a prominent increase in fibroblast cells (3(b)) and blood vessels (3(c)) as well as a reduction in inflammatory cells (3(d) by day 14. These results were compared with normal rats treated with blank films. Diabetic rats with blank film treatments revealed disorganized structures containing lots of inflammatory cells and fever fibroblast cells as well as a lack of blood vessels.

### Nitric oxide (NO) scavenging activity of VCN-2 film on wound tissues

**Fig. 4:**
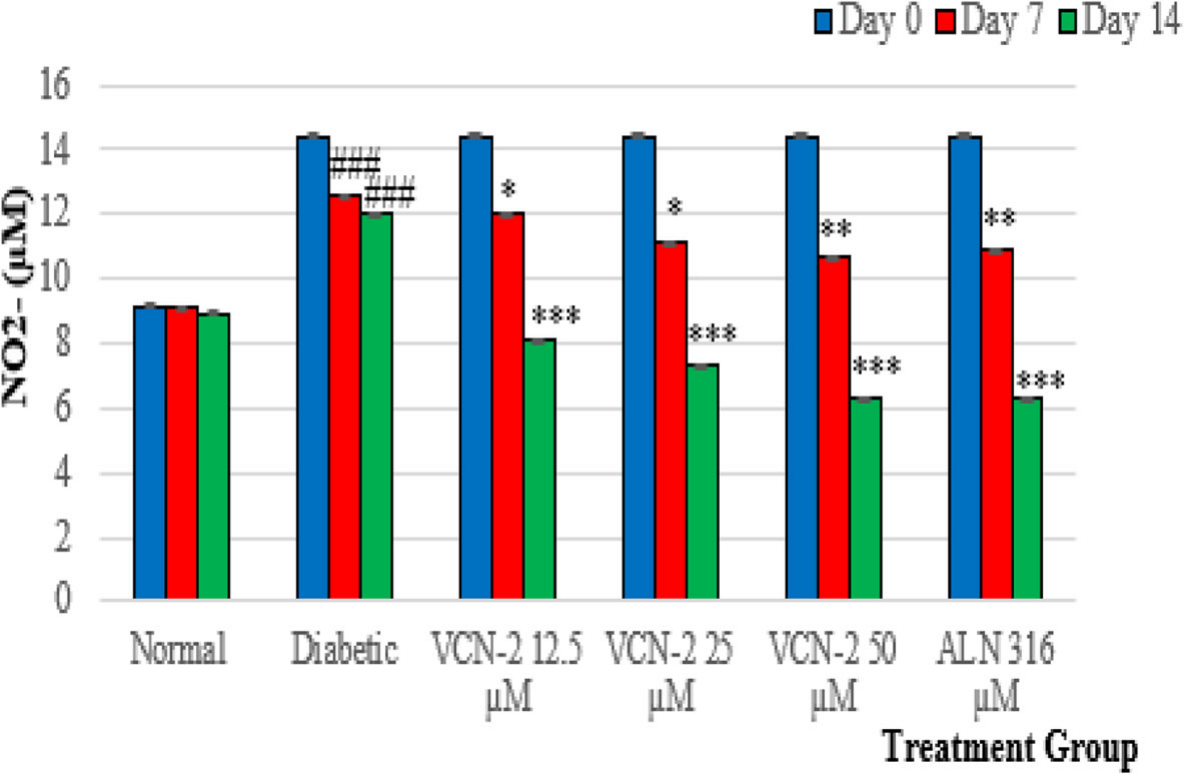
Nitrite levels. On 0, 7, and 14 postoperative days, animals were sacrificed and samples of excisional wounds were removed to determine nitrite levels by Griess reaction. Data are mean of three independent experiments and are expressed as mean ± SEM of nitric oxide levels (μM). ###*p* < 0.001 indicated statistical difference diabetic group compared to normal group; * *p* < 0.05, ** *p* < 0.01 and *** *p* < 0.001 indicated treatments groups with respect to diabetic group. (*n* = 6 animals/group/time point/experiment, ANOVA-Tukey test)

On day 14, the level of nitric oxide (NO) was significantly upregulated in the wound tissues of diabetic groups (12.05 ± 0.010 μM) compared to the normal control groups (8.95 ± 0.001 μM). There was a reduction of NO levels for the blank dressing-treated normal rats on day 7 and day 14, but these changes were insignificant. On day 14, NO levels in the diabetic rats significantly declined after treatment with VCN-2 hydrocolloid film, similar to the positive control group (6.35 ± 0.010 μM). There was a significant reduction of NO levels (6.36 ± 0.014 μM) after treatment with 50 μM VCN-2 film (Fig. 4).

### Effect of VCN-2 film on the production of inflammatory cytokines (IL-1β, IL-6 and TNF-α) and healing growth factors (VEGF and TGF-β) of wound tissue

**Fig. 5:**
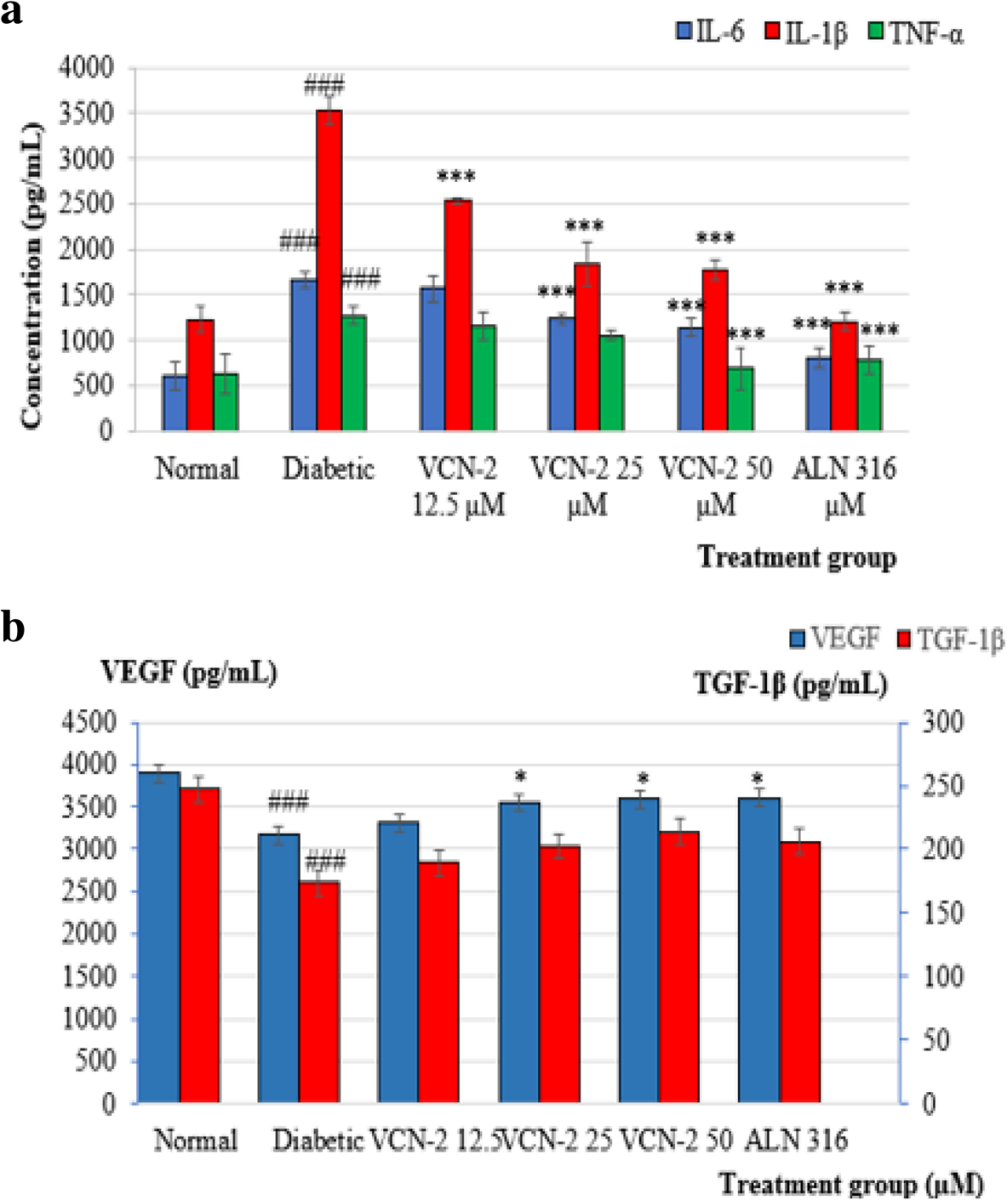
**a** Effect of VCN-2 hydrocolloid film which was topically applied on normal and diabetic rats inflicted with excision wound for 14 days. Production of pro-inflammatory cytokines such as IL-1β, IL-6 and TNF-α after different concentration of VCN-2 treatments. **b** Secretions of growth factors like VEGF and TGF-1β on day 14 with treatment of VCN-2 in concentration 12.5, 25, 50 μM. All values are expressed as mean ± SEM. Each group consists of six rats. ### *p* < 0.001, diabetic group compared to normal group; ***p < 0.001, treatments groups compared to diabetic group

As shown in Fig. 5 (a), wound tissues from the normal group expressed low level of pro-inflammatory cytokines on day 14 post-wounding. In diabetic rats, the level of inflammatory cytokines was found to be significantly elevated with IL-1β (3531 ± 159 pg/mL), IL-6 (1655 ± 88 pg/mL), and TNF-α (1271 ± 95 pg/mL). Treatment with VCN-2 produced a dose dependent reduction of pro-inflammatory cytokines in wounded tissues. Treatment with 25–50 μM VCN-2 showed significant reductions of IL-1β, IL-6 and TNF-α. Cytokine levels were found to be similar to those seen in wounds treated with alantoin.

Rat growth factors changes throughout this study are summarized in Fig. 5 (b). In general, an increase in growth factor production was observed following treatment with increased doses of VCN-2 film 14 days post-wounding. The wound tissues from the normal group showed 3896 ± 269 pg/mL VEGF and 247 ± 20 pg/mL TGF-1β on day 14. However, there were significant reductions in the production of both growth factors in the wound samples of STZ induced rats, with 3168 ± 130 pg/mL VEGF and 174 ± 31 pg/mL TGF-1β. Treatment with VCN-2 hydrocolloid film at 50 μM effectively enhanced the synthesis of VEGF (3596 ± 244 pg/mL) and TGF-1β (214 ± 25 pg/mL) in wound samples of STZ-induced rats as compared to 12.5 and 25 μM VCN-2. Likewise, the allantoin hydrocolloid films that acted as a positive control also revealed a significant increase in both growth factors in wounded tissues.

### Impact of VCN-2 film on expression of wound mediators (iNOS, COX-2, NF-κB, MMP-9, anti-HIF1α, VEGF and TGF-β)

**Fig. 6:**
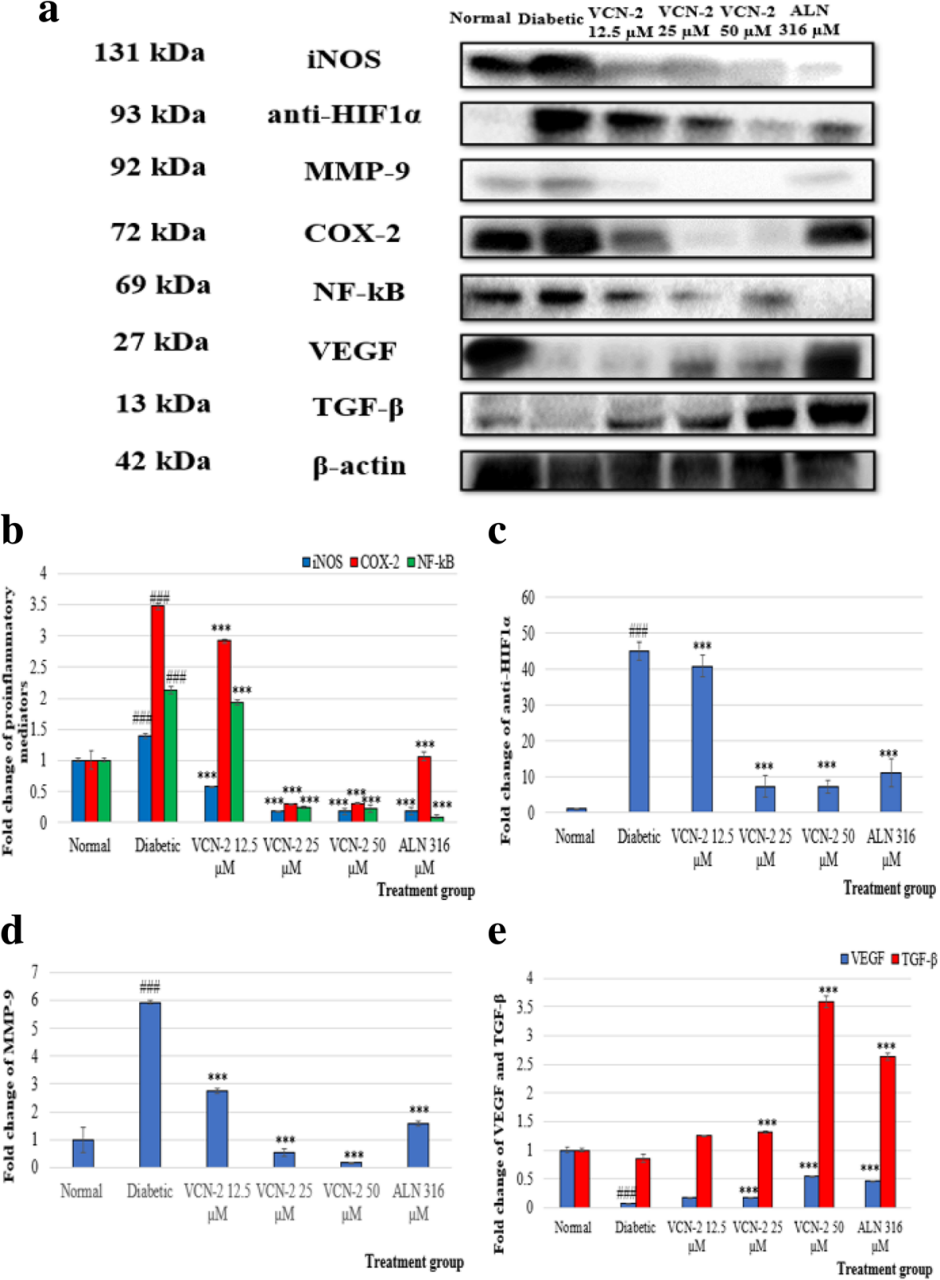
**a** Effect of VCN-2 hydrocolloid film on expression of mediators after 14 days treatment was detected by Western blotting. Down-regulation of various anti-inflammatory and up-regulation of expression of several wound healing markers in skin tissues were observed. β-actin acted as control marker. **b** Effect of VCN-2 film on expression of mediators after 14 days topically treatment was detected by immunoblotting. A graph represented densitometry analysis results of the effect of VCN-2 on proteins expression proinflammatory mediators (**c**) anti-HIF1α, (**d**) MMP-9, and (**e**) VEGF and TGF-β. All data are expressed as mean ± SEM. ### *p* < 0.001 represented statistical difference diabetic group compared to normal group; *** *p* < 0.001 represented statistical difference treatment groups compared to the diabetic group

Western blot analysis of iNOS, COX-2, NF-κB, MMP-9, anti-HIF1α, VEGF, and TGF-β proteins in wounded samples from each group are demonstrated in Fig. 6 (a). The expression of pro-inflammatory mediators such as iNOS, COX-2, and NF-κB (Fig. 6 (b)) together with anti-HIF1α and MMP-9 (Fig. 6 (c,d)) mediators were up-regulated in diabetic samples as compared to the normal samples. In contrast, the expressions of VEGF and TGF-β were down-regulated in diabetic samples as compared to the normal samples ((Fig. 6 (e)). In this study, VCN-2 treatment effectively ameliorated the expression of proinflammatory, MMP-9, and anti-HIF1α mediators (Fig. 6 (b,d)). In contrast, growth factors such as VEGF and TGF-β (Fig. 6 (e)) were enhanced after 14 days of VCN-2 treatment.

## Discussion

Vicenin-2 is a type of flavonoid glycosides that is derived from various natural plants. There are several studies on the efficiency of flavonoid glycoside in a wide variety of pharmacological activities, including anti-inflammation and wound healing [12, 28]. Pang et al. [29] supported that the expression of biomarkers such as VEGF and TGF-β accelerated wound healing, which is attributed to the presence of flavonoids. Considering the therapeutic properties of VCN-2, in vivo tests were employed to observe the efficacy of VCN-2 hydrocolloid films on wound excision in hyperglycemic rats to reveal the underlying action mechanism of VCN-2 on wound healing.

Measuring blood glucose levels is one of the most effective ways to diagnose and monitor the development of diabetes. Hence, blood glucose levels were chosen as the main disease progression parameter in this study. 50 mg/kg STZ significantly elevated blood glucose levels in fasting rats (Table 3) and created diabetic-like syndromes. This is because STZ is a diabetogenic agent that damages the DNA of pancreatic β cells by inducing necrosis [30]. This effectively reduces the synthesis of insulin in treated rats, which eventually gave rise to a diabetic phenotype (Table 3). This finding is supported by Monera [31], who successfully induced diabetes in rats with low insulin levels and high glucose levels using STZ.

Topical administration of hydrocolloid films containing VCN-2 (12.5–50 μM) on diabetic wounds gradually increased host insulin levels (Table 3). One explanation for this is that the action of VCN-2, which was released from the hydrocolloid film dressing into the wound and then the bloodstream, can increase host insulin production and the insulin sensitivity of somatic cells. Previous studies have also reported similar observations, as VCN-2 isolated from *Artemisia capillaris* was able to enhance insulin production and sensitivity [8]. However, further investigation is required to confirm the precise mechanisms of how insulin is released from β cells after VCN-2 administration.

It was observed that rat blood glucose levels were lower during wound infliction compared to the initial level (Table 3). It is speculated that the presence of certain homeostasis control mechanisms, such as negative feedback loops, exists to balance deviating glucose levels during wound infliction [32], which caused the observed behaviour. Nonetheless, rat glucose levels in this study were found to be higher than 11 mmol/L [33], suggesting that the rats were still in a diabetic state during wound infliction.

A reduction in body weight (Table 3) was observed in rats after STZ administration. This could be attributed to polyuria, which is one of the characteristics exhibited by hyperglycemia, due to excess calories being lost in urine [34]. According to Miao et al. [35], polyphagia and polydipsia in rats are hallmarks of hyperglycemia. A positive correlation between changes in pellet and fluid intakes were observed in rats after STZ injection. Consistently, results showed an increased in food and water intake (Table 3) in diabetic rats compared to normal rats. Both characteristics are clinical manifestations of diabetics in rats.

Following the topical application of VCN-2-containing hydrocolloid film, the gross wound size of diabetic rats (Fig. 1) was found to be significantly reduced and was accompanied by dose-dependent wound contraction (Table 4). This suggests that VCN-2 is effective in promoting wound healing in hyperglycemic rats. Similar findings were reported in our previous studies, where topical application of *M. oleifera* aqueous extract comprised of VCN-2 enhanced the healing of excision wounds in diabetic rats through improvements in wound contraction and reductions in wound size [14, 21].

Acceleration of complete wound closure was observed following the topical administration of VCN-2 film on wounded sites (Fig. 2(a)). Re-epithelialization of the skin was initiated upon injury. Subsequently, growth factors were released by cells to induce proliferation and migration of keratinocytes, macrophages, and fibroblast cells into the wound space [36, 37]. Interestingly, Fig. 2(b) displays notable epithelialization in diabetic wounds after VCN-2 film treatments. Granulation tissue consists of various cells, and vascular capillaries and loose connective tissues in its stroma are usually produced to fill up the injured space [38, 39]. Similar to the epithelialization process, Fig. 2(c) also suggests accelerated wound healing through the formation of granulation tissues on diabetic skin tissue.

Yang et al. [40] reported that fibroblast and blood vessel proliferations were compromised in diabetic wounds, resulting in impaired wound healing. The results in Fig. 3(a) demonstrate that VCN-2 treatments enhanced wound healing for rats in hyperglycemia conditions through fibroblast proliferation and angiogenesis. Meanwhile, higher wound recovery rates supported by fibroblast proliferation are shown in Fig. 3(b). Our results showed that fibroblast proliferation is one of the earliest events in the development of granulation tissue [41, 42].

New blood capillary formation in wound sites is crucial to the delivery of oxygen, cytokines, and essential nutritients to cells as well as the removal of waste products from proliferating cells [43]. Figure 3(c) shows a marked increase in angiogenesis in diabetic wounds treated with VCN-2 film. These findings are in accordance with Kant et al. [44], who observed that curcumin enhanced wound healing by stimulating fibroblast proliferation and promoting new blood vessel formation in diabetic rats.

Diabetic wounds showed sustained production of inflammatory cells in wound sites, which limited the effectiveness of wound closure [45]. Our data demonstrated reduction of inflammatory cells in diabetic wound sites treated with VCN-2 film (Fig. 3(d). This is consistent with Moura et al. [46], who reported that the application of neurotensin loaded collagen enhanced the diabetic wound healing of mice by reducing the creation of inflammatory cells in the wound site.

Nitrogen Oxide (NO) was detected in the wounds of normal rats. However, NO levels were significantly higher in the wounds of diabetic rats. An optimum release of NO can enhance wound healing, but the overproduction of NO over an extended period will lead to the formation of sustained chronic wounds. NO is not only a mediator involved in the inflammation of cells (macrophages, fibroblasts, and keratinocytes), but it also plays an important role in intercellular communication to modulate cell proliferation, collagen development, and wound restoration [47]. In this study, a concentration dependent decrease of NO in diabetic wound lysates after treatment with VCN-2 (Fig. 4) was observed. Our results are similar to Badr et al. [48], who successfully promoted diabetic excisional wound healing in mice by suppressing NO synthesis with bee venom treatments.

Previous studies have reported that the production of pro-inflammatory cytokines such as IL-1β, IL-6, and TNF-α as well as inflammatory cells were elevated in chronic wounds [48–50], and that VEGF and TGF-β secretion from injury cells, such as macrophages and fibroblasts, were reduced in diabetic wounds [3, 51]. In this study, VCN-2 film treatment alleviated these conditions in diabetic wounds, as the production of pro-inflammatory cytokines such as IL-1β, IL-6, and TNF-α were significantly downregulated in a dose dependent manner (Fig. 5(a)). Upregulation of growth factors such as VEGF and TGF-1β (Fig. 5(b)) were also observed in the skin wound lysate of rats in hyperglycemia conditions that were treated with VCN-2 film.

In this study, impared wound healing in diabetic rats is correlated and associated with the dysregulation of pro-inflammatory mediators and growth factor gene expressions (Fig. 6(a)). Adequate expression of proinflammatory mediators is important in producing cytokines to recruit neutrophils and removing bacteria and other contaminations from the wound site [36]. However, the prolonged expression of these cytokines is undesired as it can lead to the development of chronic wounds. Following treatment with VCN-2 films, pro-inflammatory mediators such as iNOS and COX-2 were reduced together with the gene regulator NF-κB (Fig. 6(b)), further proving VCN-2’s effectiveness in treating diabetic wounds. These results are supported by a previous study where the application of celecoxib on pressure ulcers diminished the expression of iNOS and COX-2 and enhanced wound healing by preventing prolonged inflammation [52]. In addition, similar results were reported by Dasu and Jialal, [53], who found that NF-κB transcription factor activity was reduced in diabetic toll-like receptor-4 knockout mice, which promoted wound repair. Our results suggest that the inflammation phase of delayed wound healing may be mediated by the anti-inflammatory effects exhibited by VCN-2.

VCN-2 film application resulted in a downregulation of anti-HIF1α expression in all wounds as shown in Fig. 6(c). As the upredulation of HIF1α may accelerate diabetic wound restoration, our results suggest the remedial effect of VCN-2 in wound healing. HIF1α is a transcription factor that is vital to improving angiogenesis and cell mortality during wound healing [54, 55]. Yang et al. [40] and Liu et al. [56] are consistent with our findings. Both studies reported that thioredoxin administration was able to overcome hyperglycemia-induced HIF-1α reduction and upregulated HIF-1α levels, which eventually enhanced wound healing by increasing blood supply to the wound site.

The role of Metalloproteinase (MMP) in wound healing is to degrade and remove damaged Extracellular Matrixes (ECM) to regulate a balanced composition of ECM that supports wound restoration [57]. Several studies reported that the overexpression of MMP-9 was observed in delayed wound healing [58–60]. Our results showed a decline in gelatinases MMP-9 levels following VCN-2 treatments on diabetic skins (Fig. 6(d)). Another study found that the inhibition of MMP-9 in diabetic mice promoted the re-epithelialization of excision wounds during the proliferation phase, which is similair to our findings [61].

Figure 6(e) indicatess that the activation of VEGF markers was facilitated by the expression of HIF-1α following VCN-2 treatment. VEGF is an angiogenetic factors (HIF-1α) that is regulated to promote angiogenesis and revascularization [62]. In this study, VCN-2 accelerated diabetic wound healing through the VEGF signalling pathways. Similarly, Zhou et al. [63] revealed that the angiogenesis of diabetic rat skin ulcers was accelerated by curcumol via VEGF pathways.

Figure 6(e) demonstrates an increase in TGF-β protein expression in a dose-dependent manner after VCN-2 film application. TGF-β plays an important role in wound healing by directing inflammatory cells into the wound site during the inflammation phase [64]; accelerating ECM deposition and granulation tissue formation during the proliferation phase [65]; and acceerating the expedition of collagen type 1 to replace collagen type 3 during the remodeling phase [66]. Our immunoblotting results highlighted the effectiveness of VCN-2 in enhancing diabetic wound healing, most liklely through TGF-β signalling pathways. This finding is supported by Mori et al. [67], who demonstrated that the topical application of lavender oil can enhance wound healing by promoting granulation tissue formation via TGF-β signalling pathways.

## Conclusion

**Fig. 7:**
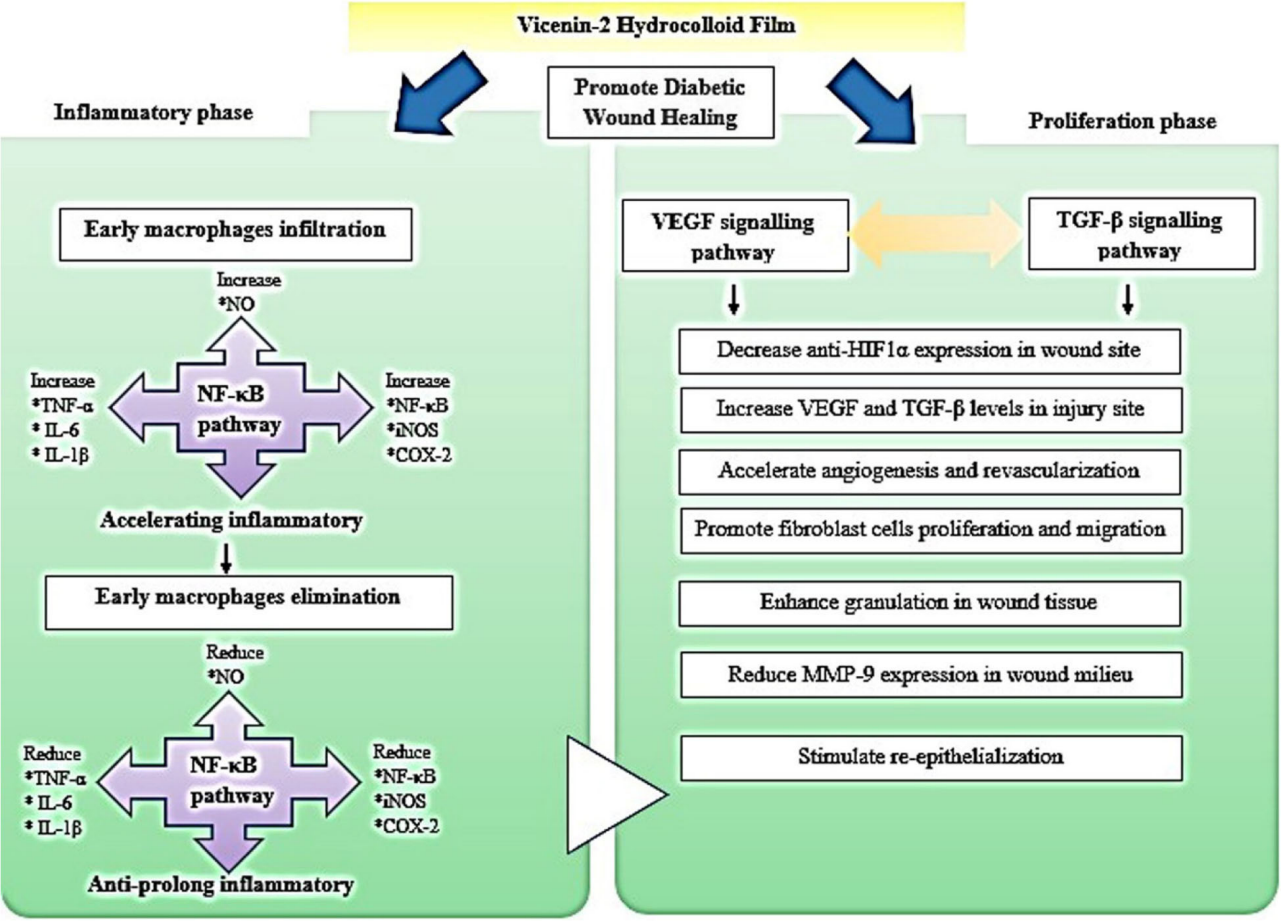
Mechanism pathways of VCN-2 to enhance excision wound healing in diabetic rats. Abbreviation: Cyclooxygenase2 (COX-2), inducible nitric oxide synthase (iNOS), inhibitor of *κ*B (I*κ*B-*α*), nitric oxide (NO), nuclear factor-kappa B (NF-*κ*B), tumor necrosis factor-alpha (TNF-*α*), interleukin (IL), metalloproteinase (MMP), hypoxia inducible transcription factor-1 alpha (HIF-1α), vascular endothelial growth factor (VEGF) and transforming growth factor beta (TGF-β)

Wound treatment using VCN-2 film effectively enhanced the healing process in hyperglycaemic rats through the anti-inflammatory effect of NF-κB signalling pathways, as suggested by a reduction of associated mediators (iNOS and COX-2), cytokines (IL-1β, IL-6 and TNF-α), and NO levels. It can be concluded that diabetic wound healing was facilitated by VCN-2 through the VEGF and TGF-β signalling pathways. The assistance of VEGF and TGF-β growth factors as well as fibroblast infiltration, proliferation, and migration in wound sites efficiently mediated the injury and returned the wound to its original state. Figure 7 illustrates the reduction of anti-HIF1α and MMP protein expressions, which is crucial to re-epithelialization, granulation tissues, and angiogenesis formation during the healing of diabetic wounds.

## Abbreviations

ALN: Allantoin
ANOVA: One-way analysis of variance
br: Boundary between unhealed and healed tissue
bv: Blood vessels
C: Connective tissue
COX-2: Cyclooxygenase-2
D: Diabetic rat
e: Epithelium
ELISA: Enzyme-linked immunosorbent assay
h: Healed area with multiple layers of fibrous connective tissue
H&E: Haematoxylin and eosin
HIF-1α: Hypoxia-inducible factor 1-alpha
I: Inflammatory cells
id: Interdigitation
ie: Immature epidermis
ig: Immature granulation tissue
IL: Interleukin
iNOS: Inducible nitric oxide synthase
N: Normal rat
NaNO_2_: Sodium nitrate
NF-κB: nuclear factor kappa-light-chain-enhancer of activated B cells
NO: Nitric oxide
PAGE: SDS-polyacrylamide gel electrophoresis
SA: Sodium alginate
STZ: Streptozotocin
TGF: Transforming growth factor
TNF: Tumor necrosis factor
u: Ulcer
VCN-2: Vicenin-2
VEGF: Vascular endothelial growth factor
W: Wound
